# Impairment of Treg suppression by GITR ligation is effective selectively in the context of a pro-inflammatory tumor microenvironment

**DOI:** 10.1186/2051-1426-2-S3-P242

**Published:** 2014-11-06

**Authors:** Roberta Zappasodi, Sadna Budhu, David Schaer, Xia Yang, Hong Zhong, Taha Merghoub, Jedd D Wolchok

**Affiliations:** 1 Sloan-Kettering Institute, New York, NY, USA; 2 Memorial Sloan Kettering Cancer Center, New York, NY, USA; 3 ImClone Systems, USA

## 

The increased understanding of immunoregulatory receptor pathways is now offering promising opportunities for immunotherapeutic intervention, as exemplified by the success of anti-CTLA-4 antibody (Ab) Ipilimumab. More recently, an agonist Ab for the co-stimulatory receptor glucocorticoid-induced TNF receptor (GITR) has entered the clinical evaluation. Preclinical studies have well documented the therapeutic efficacy of GITR ligation using the agonist Ab DTA-1. However, as a monotherapy, it is no longer effective against advanced B16 melanoma. As anti-GITR immunotherapy is being translated into the clinic, understanding the mechanisms of therapeutic efficacy or failure is warranted, and will allow the identification of prognostic biomarkers and guide the design of combination therapies. To this end, we first studied the phenotypic and functional modulations of T cells, either effector (Teff) or regulatory T cells (Tregs), when co-cultured *in vitro *with B16F10 cells in the presence of DTA-1. We subsequently verified whether the *in vitro *DTA-1-induced modulations occurred at a different extent *in vivo *when tumor-bearing mice were treated in curative or refractory conditions.

*In vitro *DTA-1-preincubated Tregs lost their immunosuppressive activity without any modulation in the Treg-associated transcription factors Foxp3 and Helios. Accordingly, DTA-1-treated Treg cultures showed an increased production of IFNg, TNF-alpha, TGF-beta, IL-17 and IL-6, pointing to the promotion of a Th1/Th17 differentiation profile following GITR engagement. *In vivo *analysis of B16F10 microenvironment shows a reduction in Teff activation status, and an increase in the frequency of Tregs, as well as their expression of Foxp3, CD25 and OX40, in more advanced disease. Similarly, MHC-II was expressed at significantly lower levels in advanced tumors. Interestingly, DTA-1 treatment reduced the frequency of tumor-infiltrating Tregs and their expression of Foxp3 and Helios either in curative or refractory condition (advanced tumors). By contrast, Teff activation and tumor-associated MHC-II expression were induced only by optimal DTA-1 treatment.

Our data indicate that DTA-1 affects tumor-induced immune-tolerance independently from the stage of the disease, whereas T-cell activation and tumor immune recognition are favored selectively in the presence of low tumor burden. This provides the rationale for studying DTA-1 therapy in combination with strategies that can promote Teff functions and/or directly affect the tumor burden, such as blockade of immune checkpoints (anti-CTLA-4 and anti-PD-1 Abs), stimulation of other co-stimulatory molecules, or targeted therapies and radiation therapy.

